# Individual and combined associations of body mass index and waist circumference with components of metabolic syndrome among multiethnic middle-aged and older adults: A cross-sectional study

**DOI:** 10.3389/fendo.2023.1078331

**Published:** 2023-02-22

**Authors:** Mei Yang, Yan Zhang, Wanyu Zhao, Meiling Ge, Xuelian Sun, Gongchang Zhang, Birong Dong

**Affiliations:** ^1^ National Clinical Research Center for Geriatrics, West China Hospital, Sichuan University, Chengdu, China; ^2^ Center of Gerontology and Geriatrics, West China Hospital, Sichuan University, Chengdu, China

**Keywords:** waist circumference, body mass index, obesity phenotypes, components of metabolic syndrome, older adults

## Abstract

**Objectives:**

Body mass index (BMI) and waist circumference (WC) are closely associated with metabolic syndrome and its components. Hence, a combination of these two obesity markers may be more predictive. In this study, we aimed to investigate the individual and combined associations of BMI and WC with selected components of metabolic syndrome and explored whether age, sex and ethnicity affected the aforementioned associations.

**Methods:**

A total of 6,298 middle-aged and older adults were included. Based on BMI and WC, the participants were divided into 4 groups: comorbid obesity (BMI ≥ 28 kg/m^2^ and WC< 85/90 cm for women/men), abdominal obesity alone (BMI< 28 kg/m^2^ and WC≥ 85/90 cm for women/men), general obesity alone (BMI ≥ 28 kg/m^2^ and WC< 85/90 cm for women/men) and nonobesity subgroups (BMI< 28 kg/m^2^ and WC< 85/90 cm for women/men). Selected components of metabolic syndrome were evaluated using the criteria recommended by the Chinese Diabetes Society. Poisson regression models with robust variance were used to evaluate the associations of obesity groups with selected components of metabolic syndrome. An interaction test was conducted to explore whether age, sex and ethnicity affect the aforementioned associations.

**Results:**

Compared with participants in the reference group (comorbid obesity), participants in the other 3 groups showed a decreased prevalence of fasting hyperglycemia (PR=0.83, 95% CI=0.73–0.94 for abdominal obesity alone, PR=0.60, 95% CI=0.38–0.96 for general obesity alone and PR=0.46, 95% CI=0.40–0.53 for nonobesity), hypertension (PR=0.86, 95% CI=0.82–0.90 for abdominal obesity alone, PR=0.80, 95% CI=0.65–0.97 for general obesity alone and PR=0.69, 95% CI = 0.66–0.73 for nonobesity) and hypertriglyceridemia (PR=0.88, 95% CI=0.82–0.95 for abdominal obesity alone, PR=0.62, 95% CI=0.47–0.81 for general obesity alone and PR=0.53, 95% CI=0.49–0.57 for nonobesity). However, participants in the abdominal obesity alone and nonobesity groups showed a decreased prevalence of low HDL-C levels while participants in the general obesity alone group did not (PR=0.65, 95% CI=0.41–1.03, p>0.05). In addition, the aforementioned associations were not affected by age, sex or ethnicity (all p for interactions>0.05).

**Conclusions:**

Comorbid obesity is superior to general and abdominal obesity in identifying individuals at high risk of developing metabolic syndrome in middle-aged and older adults. Great importance should be attached to the combined effect of BMI and WC on the prevention and management of metabolic syndrome.

## Introduction

Metabolic syndrome is a health-threatening public health issue characterized by a combination of metabolic abnormalities including abdominal obesity, fasting hyperglycemia, hypertension, hypertriglyceridemia and reduced high-density lipoprotein cholesterol (HDL-C) levels. It is highly prevalent in China. A meta-analysis of 35 observational studies with 226,653 participants aged 15 years and over reported that the prevalence of metabolic syndrome in mainland China was 24.5% (1). Metabolic syndrome and its components are significantly influenced by sex, age, and ethnicity. A previous study of 3,423 adults showed that individuals aged 40–59 years had a threefold increased prevalence of metabolic syndrome compared with those aged 20–39 years (2). Furthermore, male participants aged 60 years and over had a fourfold increased prevalence of metabolic syndrome compared with female participants of the same age. Non-Hispanic white males, non-Hispanic black and Mexican-American females had a higher prevalence of metabolic syndrome than the other participants. Metabolic syndrome and its components have been well accepted as important risk factors for cardiovascular diseases (3), which are the leading causes of disability and death in China (4). Therefore, early identification of modifiable risk factors for metabolic syndrome has substantial importance.

Obesity is a serious health problem worldwide. When obesity is classified on the basis of BMI ≥ 30, it affects approximately 13% of adults worldwide (5). Almost 85 million adults aged 18–69 years in China suffer from obesity in 2018 (6). Individuals with obesity are at increased risk of developing hypertension (7), dyslipidemia (8) and glucose intolerance (9).

Currently, body mass index (BMI) or waist circumference (WC) are the most frequently used measurements for obesity (10). Both BMI and WC have been associated with metabolic syndrome and its components. However, the priority of these two measurements in identifying individuals at high risk of developing metabolic syndrome remains controversial. A previous study of 14,924 adults from the USA reported that obesity-related health risks were attributed to WC rather than BMI (11). However, Yang et al. reported that BMI is a better predictor than WC for type 2 diabetes mellitus (12) and hypertension (13) in older adults. These conflicting results of previous studies suggest the need for better markers. In addition, the body fat distribution is significantly changed with aging, which limits the usefulness of a single marker to identify individuals at high risk of metabolic syndrome across all age groups. As a result, evaluating obesity and its related comorbidities based on BMI or WC may underestimate health risks.

Although BMI and WC are highly correlated with each other (14), increased BMI may not always be accompanied by an increased WC and the vice versa (15). Considering the possibility that there exist unique characteristics of BMI and WC, a combination of BMI and WC might enhance the predictive value for metabolic syndrome and its components, which could expedite and simplify the screening process for individuals at high risks.

Although previous studies have reported that individuals with both increased BMI and increased WC are more prone to incident hypertension (16), stroke (17), cardiovascular diseases (18) and cognitive impairment (19), there exists substantial variation between obesity markers and disease development due to difference in age, sex and ethnicity (20).

The aim of our study was to evaluate the individual and combined associations of BMI and WC with selected components of metabolic syndrome in middle-aged and older adults and to explore whether age, sex and ethnicity affected the aforementioned associations.

## Methods

### Study design and participants

This cross-sectional study analyzed the data from 6,298 participants of the West China Health and Aging Trend (WCHAT) study. The details of the WCHAT study have been published previously (21). The WCHAT study recruited 7,536 community-dwelling individuals aged 50 years and over from Sichuan, Yunnan, Guizhou, and Xinjiang provinces in 2018. The demographic information of the participants was collected by trained interviewers *via* in-person interviews. Additionally, the participants underwent physical examination, anthropometric measurements, and blood tests.

The current study included participants with available data for BMI, WC, and one of the following: blood pressure, fasting plasma glucose level, triglyceride level, and HDL-C level. This study was approved by the Ethics Committee of West China Hospital, Sichuan University (reference no.: 2017-445). Informed consent was obtained from all participants.

### Data collection

We performed in-person interviews to collect information regarding sociodemographic characteristics including age, sex, educational background (illiterate, primary school, or secondary school or above), ethnicity (Han, Qiang, Tibetan, Yi, Uighur, or other ethnic minorities), marital status (married or single), smoking status (ever smokers [for > 6 months] or never smokers), alcohol consumption, hypertension, and diabetes.

### Anthropometric measurements and blood test

With the participants barefoot and wearing light clothes, stadiometer and weighing scale (Tsinghua tongfang, Beijing, China) were used to measure their height and weight, respectively. A soft tape was used to measure the waist at the level of the navel by trained volunteers (22). The waist circumference was measured twice and the average value was recorded. BMI was calculated as weight divided by height in meters squared. Before blood pressure measurement, the participants were asked to rest in a seated position for 5–10 mins. The blood pressure was measured twice using an electronic sphygmomanometer (Yuwell, Jiangsu, China) and the average measurement was recorded. Blood samples were collected from each participant in the morning after at least 10 h of fasting. Fasting glucose, triglycerides and high-density lipoprotein cholesterol (HDL-C) were measured with an automatic biochemical analyzer (OLympus Au400, Japan).

### Obesity subgroups

Based on criteria validated for the Chinese population, obesity was defined as BMI ≥ 28 kg/m^2^ or WC≥ 85 cm for women and WC≥ 90 cm for men (23, 24). In our study, participants were further divided into 4 groups: comorbid obesity (BMI ≥ 28 kg/m^2^ and WC< 85/90 cm for women/men), abdominal obesity alone (BMI< 28 kg/m^2^ and WC ≥ 85/90 cm for women/men), general obesity alone (BMI ≥ 28 kg/m^2^ and WC< 85/90 cm for women/men) and nonobesity subgroups (BMI<28 kg/m^2^ and WC< 85/90 cm for women/men).

### Assessment of selected components of metabolic syndrome

The criteria recommended by the Chinese Diabetes Society (25) were used to diagnose hypertriglyceridemia (triglyceride level≥1.70 mmol/L), reduced HDL-C (HDL-C level<1.04 mmol/L), hypertension (systolic blood pressure≥130 mmHg, diastolic blood pressure≥ 85 mmHg, or use of antihypertensive medications), and hyperglycemia (fasting plasma glucose level ≥6.1 mmol/L or use of antidiabetic medications).

### Statistical analysis

Stata software (version 14.1; Stata Corp, College Station, TX, USA) was used for data analysis. The normal distribution of the data was analyzed with the Shapiro-Wilk test. Continuous data were expressed as the means ± standard deviations or medians with interquartile range. Categorical data were presented as counts (percentages). The differences between groups were tested using analysis of variance (ANOVA) or the Kruskal-Wallis test for normally distributed or skewed continuous variables. The chi-square test was used to analyze the categorical variables. Poisson regression with robust variance was adopted to explore the associations of different obesity subgroups with selected components of metabolic syndrome. Prevalence ratios (PRs) and 95% confidence intervals (CIs) were used to present the results. The confounding variables included age, sex, ethnicity, educational status, marital status, smoking, and alcohol consumption. Furthermore, to explore whether the aforementioned associations were affected by age, sex and ethnicity, participants were further divided according to sex (male/female), age (50–59 years/over 60 years) and ethnicity (Han/Qiang/Tibetan/Yi/Uighur) and an interaction test was conducted. P<0.05 was considered statistically significant.

## Results

### Baseline characteristics of participants

The study included 6,298 individuals (mean age=62.3 ± 8.1 years; 63.02% females). Participants with comorbid obesity were the youngest among the 4 groups and accounted for 21.9% of the participants. The majority of participants was Han Chinese, followed by Qiang, Tibetan, Yi, Uighur, and other ethnic minorities. Significant differences were observed among the 4 groups in terms of age, sex, ethnicity, smoking status, BMI, and WC. Participants with comorbid obesity showed a higher prevalence of hyperglycemia, hypertriglyceridemia, hypertension, and reduced HDL-C levels than other groups. The baseline characteristics of the participants are presented in **Table 1**.

**Table 1:** Characteristics of study participants by obesity subgroups.

	WC < 90 (men) or 85 (women) cm		WC ≥ 90 (men) or 85 (women) cm		P value
	BMI < 28.0 kg/m^2^	BMI ≥ 28.0 kg/m^2^	BMI < 28.0 kg/m^2^	BMI ≥ 28.0 kg/m^2^	
	Nonobesity (n = 2,852)	General obesity alone (n = 96)	Abdominal obesity alone (n = 1,965)	Comorbid obesity (n = 1,385)	
Age, years ^†^	62.9 ± 8.5	62.1 ± 8.4	62.4 ± 8.0	61.0 ± 7.6	< 0.001
Female, n (%)	1618 (56.7)	58 (60.4)	1352 (68.8)	941 (67.9)	< 0.001
Education, n (%)					
Illiteracy	808 (28.4)	25 (26.0)	530 (27.0)	388 (28.1)	0.160
Primary school	997 (35.1)	29 (30.2)	659 (33.6)	440 (31.8)	
Secondary school and above	1037 (36.5)	42 (43.8)	774 (39.4)	554 (40.1)	
Ethnicity, n (%)					
Han	1185 (41.5)	14 (14.6)	723 (36.8)	385 (27.8)	< 0.001
Qiang	459 (16.1)	4 (4.2)	485 (24.7)	296 (21.4)	
Tibetan	519 (18.2)	22 (22.9)	321 (16.3)	361 (26.1)	
Yi	314 (11.0)	5 (5.2)	134 (6.8)	72 (5.2)	
Uighur	68 (2.4)	37 (38.5)	197 (10.0)	205 (14.8)	
Other ethnic minorities*	307 (10.8)	14 (14.6)	105 (5.3)	66 (4.8)	
Marital status, n (%)					
Married	2379 (83.7)	75 (78.1)	1653 (84.2)	1143 (82.7)	0.330
Single	463 (16.3)	21 (21.9)	310 (15.8)	239 (17.3)	
History of smoking, n (%)	666 (23.6)	25 (26.0)	299 (15.3)	187 (13.6)	< 0.001
History of alcohol consumption, n (%)	762 (26.9)	20 (20.8)	503 (25.6)	326 (23.6)	0.095
BMI, kg/m^2 ‡^	22.7 (21.1–24.3)	29.7 (28.6–32.4)	25.7 (24.3–26.8)	30 (28.9–31.7)	< 0.001
WC, cm ^‡^	80 (75–83.3)	83 (80–84.8)	91.7 (88.7–95.5)	99 (94–104)	< 0.001
Components of metabolic syndrome, n (%)					
Fasting hyperglycemia	368 (12.9)	15 (15.6)	419 (21.3)	338 (24.4)	< 0.001
Hypertriglyceridemia	831 (29.2)	34 (35.4)	923 (47.1)	718 (52.0)	< 0.001
Low HDL-C level	435 (15.3)	14 (14.6)	406 (20.7)	382 (27.6)	< 0.001
Hypertension	1234 (55.5)	42 (60.9)	1033 (65.9)	821 (74.6)	< 0.001

^†^ Data are presented as the means ± standard deviations (SD).

^‡^ Data are presented as the median and interquartile range (IQR).

* The other ethnic minorities included 13 ethnicities, each with 1–172 participants.

BMI, body mass index; WC, waist circumference; G, group; HDL-C, high-density lipoprotein cholesterol.

### Associations of different obesity groups with selected components of metabolic syndrome

The results of Poisson regression analysis with robust variance were presented in **Table 2**. Comorbid obesity was selected as the reference. Compared with the reference group, participants in the other 3 groups had a lower prevalence of fasting hyperglycemia (PR=0.83, 95% CI=0.73–0.94 for abdominal obesity alone, PR=0.60, 95% CI=0.38–0.96 for general obesity alone and PR=0.46, 95% CI=0.40–0.53 for nonobesity), hypertension (PR=0.86, 95% CI=0.82–0.90 for abdominal obesity alone, PR=0.80, 95% CI=0.65–0.97 for general obesity alone and PR=0.69, 95% CI=0.66–0.73 for nonobesity), and hypertriglyceridemia (PR=0.88, 95% CI=0.82–0.95 for abdominal obesity alone, PR=0.62, 95% CI=0.47–0.81 for general obesity alone and PR=0.53, 95% CI=0.49–0.57 for nonobesity) after adjustment for confounders. However, in the fully adjusted model, participants in the abdominal obesity alone and nonobesity groups showed a decreased prevalence of low HDL-C levels while participants in the general obesity alone group did not (PR=0.65, 95% CI=0.41–1.03, p>0.05)

**Table 2:** Associations of obesity subgroups with individual components of metabolic syndrome.

Cases/Controls	Comorbid obesity	Abdominal obesity alone	General obesity alone	Nonobesity
		PR [95% CI]	PR [95% CI]	PR [95% CI]
Fasting hyperglycemia vs non fasting hyperglycemia(reference)				
(1,140/5,158)				
Crude	Ref.	0.87 (0.77–0.99) *	0.64 (0.39–1.02)	0.52 (0.46–0.60) **
Adjusted model	Ref.	0.83 (0.73–0.94) *	0.60 (0.38–0.96) *	0.46 (0.40–0.53) **
Hypertension vs nonhypertension (reference)				
(3,130/1,829)				
Crude	Ref.	0.88 (0.84–0.92) **	0.81 (0.67–0.98) *	0.74 (0.70–0.78) **
Adjusted model	Ref.	0.86 (0.82–0.90) **	0.80 (0.65–0.97) *	0.69 (0.66–0.73) **
Hypertriglyceridemia vs nonhypertriglyceridemia (reference)				
(2,506/3,778)				
Crude	Ref.	0.9 (0.82–0.94) *	0.68 (0.51–0.89) *	0.56 (0.51–0.60) **
Adjusted model	Ref.	0.88 (0.82–0.95) **	0.62 (0.47–0.81) **	0.53 (0.49–0.57) **
Low HDL-C level vs normal HDL levels (reference)				
(1,237/5,051)				
Crude	Ref.	0.74 (0.66–0.84) **	0.52 (0.32–0.86) *	0.55 (0.48.0.62) **
Adjusted model	Ref.	0.74 (0.66–0.83) **	0.65 (0.41–1.03)	0.44 (0.39–0.50) **

Cases and controls refer to the numbers of participants with/without fasting hyperglycemia, hypertension, hypertriglyceridemia or low HDL-C levels.

PR, prevalence ratio; CI, confidence interval; HDL, high-density lipoprotein cholesterol.

Adjusted model: adjusted for age, sex, ethnicity, education, marital status, smoking status, and alcohol consumption.

*p < 0.05; **p < 0.001.

Our findings indicated that individuals with comorbid obesity might be at higher risk of developing metabolic syndrome than other obesity groups. In addition, the aforementioned associations were not affected by sex, age or ethnicity in middle-aged and older adults of western China. (all p for interactions>0.05) (**Tables 3**–**6**)

**Table 3:** Associations of obesity subgroups with fasting hyperglycemia in the adjusted model.

	Comorbid obesity	Abdominal obesity alone	General obesity alone	Nonobesity	P for interaction
		PR [95% CI]	PR [95% CI]	PR [95% CI]	
Overall	Ref.	0.83 (0.73–0.94) *	0.60 (0.38–0.96) *	0.46 (0.40–0.53) **	
**Sex ^a^**					0.762
Male	Ref.	0.81 (0.67–0.98) *	0.99 (0.57–1.70)	0.42 (0.35–0.51) *	
Female	Ref.	0.86 (0.72–1.02)	0.31 (0.12–0.77) *	0.51 (0.42–0.62) **	
**Age ^b^**					0.913
50–59 years	Ref.	0.83 (0.66–1.02)	0.23 (0.06–0.89) *	0.39 (0.30–0.50) **	
≥ 60 years	Ref.	0.85 (0.73–1.00)	0.85 (0.50–1.37)	0.51 (0.43–0.60) **	
**Ethnicity ^c^**					0.359
Han	Ref.	0.86 (0.69–1.06)	0.48 (0.13–1.75)	0.47 (0.38–0.59) **	
Qiang	Ref.	0.81 (0.62–1.05)	0.81 (0.19–3.37)	0.44 (0.32–0.61) **	
Tibetan	Ref.	0.78 (0.56–1.08)	0.73 (0.26–2.06)	0.35 (0.24–0.50) **	
Yi	Ref.	0.89 (0.51–1.54)	NA	0.40 (0.24–0.66) **	
Uighur	Ref.	1.08 (0.75–1.07)	0.91 (0.44–1.90)	0.37 (0.16–0.82) *	

Nonfasting hyperglycemia as a reference.

PR, prevalence ratio; CI, confidence interval; HDL, high-density lipoprotein cholesterol.

a: adjusted for age, ethnicity, education, marital status, smoking status, and alcohol consumption.

b: adjusted for sex, ethnicity, education, marital status, smoking status, and alcohol consumption.

c: adjusted for age, sex, education, marital status, smoking status, and alcohol consumption.

*p < 0.05; **p < 0.001.

**Table 4:** Associations of obesity subgroups with hypertension in the adjusted model.

	Comorbid obesity	Abdominal obesity alone	General obesity alone	Nonobesity	P for
		PR [95% CI]	PR [95% CI]	PR [95% CI]	interaction
Overall	Ref.	0.86 (0.82–0.90) **	0.80 (0.65–0.97) *	0.69 (0.66–0.73) **	
**Sex ^a^**					0.769
Male	Ref.	0.90 (0.83–0.98) *	0.86 (0.65–1.14)	0.76 (0.70–0.82) **	
Female	Ref.	0.84 (0.79–0.89) *	0.79 (0.60–1.03)	0.66 (0.61–0.70) **	
**Age ^b^**					0.221
50–59 years	Ref.	0.79 (0.72–0.87) **	0.79 (0.55–1.13)	0.61 (0.55–0.67) **	
≥ 60 years	Ref.	0.90 (0.85–0.95) **	0.82 (0.65–1.03)	0.75 (0.71–0.80) **	
**Ethnicity ^c^**					0.072
Han	Ref.	0.87 (0.80–0.95) *	1.08 (0.77–1.51)	0.71 (0.65–0.77) **	
Qiang	Ref.	0.77 (0.70–0.85) **	1.16 (0.94–1.43)	0.61 (0.54–0.68) **	
Tibetan	Ref.	0.78 (0.69–0.87) **	0.82 (0.55–1.20)	0.67 (0.60–0.75) **	
Yi	Ref.	0.89 (0.71–1.11)	0.70 (0.27–1.77)	0.78 (0.64–0.95) *	
Uighur	Ref.	1.17 (1.00–1.38) *	0.89 (0.61–1.29)	0.76 (0.55–1.03)	

Nonhypertension as reference.

PR, prevalence ratio; CI, confidence interval; HDL, high-density lipoprotein cholesterol.

a: adjusted for age, ethnicity, education, marital status, smoking status, and alcohol consumption.

b: adjusted for sex, ethnicity, education, marital status, smoking status, and alcohol consumption.

c: adjusted for age, sex, education, marital status, smoking status, and alcohol consumption.

*p < 0.05; **p < 0.001.

**Table 5:** Associations of obesity subgroups with hypertriglyceridemia in the adjusted model.

	Comorbid obesity	Abdominal obesity alone	General obesity alone	Nonobesity	P for
		PR [95% CI]	PR [95% CI]	PR [95% CI]	interaction
Overall	Ref.	0.88 (0.82–0.95) **	0.62 (0.47–0.81) **	0.53 (0.49–0.57) **	
Sex ^a^					0.941
Male	Ref.	0.85 (0.76–0.96) *	0.68 (0.45–1.03)	0.45 (0.39–0.51) **	
Female	Ref.	0.90 (0.82–0.98) *	0.59 (0.41–0.85) *	0.59 (0.54–0.65) **	
Age ^b^					0.425
50–59 years	Ref.	0.93 (0.83,1.03)	0.56 (0.36–0.88) *	0.56 (0.50–0.63) **	
≥ 60 years	Ref.	0.85 (0.78–0.93) *	0.68 (0.49–0.96) *	0.51 (0.46–0.57) **	
Ethnicity ^c^					0.079
Han	Ref.	0.85 (0.76–0.95) *	0.80 (0.47–1.38)	0.51 (0.45–0.57) **	
Qiang	Ref.	0.90 (0.75–1.07)	0.53 (0.10–2.63)	0.49 (0.39–0.61) **	
Tibetan	Ref.	0.83 (0.68–1.01)	0.47 (0.19–1.16)	0.50 (0.41–0.62) **	
Yi	Ref.	0.86 (0.68–1.07)	0.91 (0.44–1.88)	0.52 (0.42–0.66) **	

Nonhypertriglyceridemia as reference.

PR, prevalence ratio; CI, confidence interval; HDL, high-density lipoprotein cholesterol.

a: adjusted for age, ethnicity, education, marital status, smoking status, and alcohol consumption.

b: adjusted for sex, ethnicity, education, marital status, smoking status, and alcohol consumption.

c: adjusted for age, sex, education, marital status, smoking status, and alcohol consumption.

*p < 0.05; **p < 0.001.

**Table 6:** Associations of obesity subgroups with low HDL-C levels in the adjusted model.

	Comorbid obesity	Abdominal obesity alone	General obesity alone	Nonobesity	P for
		PR [95% CI]	PR [95% CI]	PR [95% CI]	interaction
Overall	Ref.	0.74 (0.66–0.83) **	0.65 (0.41–1.03)	0.44 (0.39–0.50) **	
**Sex ^a^**					0.425
Male	Ref.	0.79 (0.68–0.92) *	0.80 (0.48–1.33)	0.44 (0.37–0.51) **	
Female	Ref.	0.71 (0.59–0.84) **	0.52 (0.22–1.22)	0.47 (0.39–0.57) **	
**Age ^b^**					0.970
50–59 years	Ref.	0.75 (0.62–0.90) **	0.56 (0.22–1.40)	0.44 (0.37–0.53) **	
≥ 60 years	Ref.	0.74 (0.63–0.87) **	0.74 (0.44–1.23)	0.45 (0.38–0.53) **	
**Ethnicity ^c^**					0.687
Han	Ref.	0.73 (0.60–0.88) *	0.40 (0.11–1.41)	0.48 (0.40–0.58) **	
Qiang	Ref.	0.66 (0.50–0.87) *	0.69 (0.10–4.55)	0.36 (0.25–0.49) **	
Tibetan	Ref.	0.61 (0.48–0.76) **	0.96 (0.58–1.59)	0.35 (0.27–0.45) **	
Yi	Ref.	1.15 (0.67–1.97)	0.81 (0.22–2.98)	0.93 (0.56–1.52)	

Normal HDL-C levels as reference.

PR, prevalence ratio; CI, confidence interval; HDL, high-density lipoprotein cholesterol.

a: adjusted for age, ethnicity, education, marital status, smoking status, and alcohol consumption.

b: adjusted for sex, ethnicity, education, marital status, smoking status, and alcohol consumption.

c: adjusted for age, sex, education, marital status, smoking status, and alcohol consumption.

*p < 0.05; **p < 0.001.

## Discussion

The main finding of our study was that comorbid obesity was superior to general or abdominal obesity alone in identifying individuals at high risk of selected components of metabolic syndrome in middle-aged and older adults. The results of our study revealed that compared with comorbid obesity, the prevalence of fasting hyperglycemia decreased by 17% for abdominal obesity alone, 40% for general obesity alone and 54% for nonobesity. The prevalence of hypertension decreased by 14% for abdominal obesity alone, 20% for general obesity alone and 31% for nonobesity. Moreover, the prevalence of hypertriglyceridemia decreased by 12% for abdominal obesity alone, 38% for general obesity alone and 47% for nonobesity. In addition, the prevalence of low HDL-C levels decreased by 26% for abdominal obesity alone, 35% for general obesity alone and 54% for nonobesity. The prevalence of selected components of metabolic syndrome decreased with a decrease in BMI and WC.

Our study indicated that comorbid obesity could augment the deleterious effects of general or abdominal obesity on the metabolic status of multiethnic middle-aged and older adults in western China. Our results were in line with the findings of previous studies on hypertension and diabetes. Momin et al. (16) found that men (odds ratio [OR]=3.10, 95% CI=1.48–6.50) and women (OR=2.51, 95% CI=1.43–4.40) with comorbid obesity had the highest prevalence of hypertension during the 2.3 years of follow-up. Furthermore, Kazuteru et al. (26) reported that a combination of overweight and abdominal obesity increased the risk of incident diabetes (OR=2.77, 95% CI=1.55–5.15). Yet, Hyunsoo et al. found that higher risks of hypertriglyceridemia (OR=3.79, 95% CI=1.75–8.22) and fasting hyperglycemia (OR=3.19, 95% CI=1.47–6.89) were reported in individuals with abdominal obesity, whereas higher risks of low HDL-C level (OR=2.33, 95% CI=1.59–3.43) and hypertension (OR=2.36, 95% CI=1.54–3.62) were reported in individuals with composite obesity (27). The discrepancy of these findings may derive from the relatively small sample size, younger age of participants, as well as the different diagnostic criteria.

As previously mentioned, there exists variation regarding associations of a single obesity marker (BMI or WC) with disease development due to difference in age, sex and ethnicity (20). However, associations between comorbid obesity and individual components of metabolic syndrome were not affected by sex, age, and ethnicity, which supports that comorbid obesity is more reliable for identifying individuals at high risk of developing metabolic syndrome.

Although BMI and WC are closely related, there exists significant variability in the body fat distribution during our lifetime. An important concept is metabolically healthy obesity, which refers to individuals with obesity who are free of other metabolic diseases (28). Presently, numerous studies have evaluated obesity and its related comorbidities by either BMI or WC. However, single obesity marker may underestimate the health risks. The combination of BMI and WC can better identify individuals at high risks of metabolic syndrome.

In addition to metabolic syndrome, individuals with both high BMI and enlarged WC have substantially increased risks of proteinuria (29), early menopause (30), cognitive impairment (19), several types of cancers (31), major cardiovascular events (32) as well as mortality (33). Therefore, particular attention should be paid to the combination of BMI and WC in clinical practice.

The main strength of our study is that we have confirmed that comorbid obesity is superior to general or abdominal obesity in identifying individuals at high risk of developing metabolic syndrome based on a relatively large sample size. Nevertheless, this study had some limitations. First, the study participants were selected from community-dwelling residents in western China. Therefore, the results cannot be generalized to individuals residing in other regions. Second, the sample size in some subgroups was relatively small, which may have affected the power of the statistical analyses. Third, we only enrolled participants aged 50 years and over and future studies should also enroll younger participants. Last, we failed to include some confounders, such as drug use and other comorbidities.

## Conclusions

Comorbid obesity is superior to general and abdominal obesity in identifying individuals at high risk of developing metabolic syndrome and its components in middle-aged and older adults. The aforementioned associations were not affected by age, sex or ethnicity. Great importance should be attached to the combined effect of BMI and WC on the prevention and management of metabolic syndrome.

## Data availability statement

The original contributions presented in the study are included in the article/supplementary material. Further inquiries can be directed to the corresponding author.

## Ethics statement

The studies involving human participants were reviewed and approved by the Ethics Committee of West China Hospital, Sichuan University. The patients/participants provided their written informed consent to participate in this study.

## Author contributions

MY and YZ contributed to the conception and design of this study. MY, YZ, GZ, and WZ contributed to the literature review. MY, XS, and WZ contributed to data analyses. WZ and MG contributed to data interpretation. MY drafted the article, while MG and BD critically appraised it and revised it. All authors contributed to the article and approved the submitted version.
